# The acute effects of a commercial pre workout product, wodFuel®, on performance of a Crossfit exercise series, the Cindy

**DOI:** 10.1186/1550-2783-11-S1-P21

**Published:** 2014-12-01

**Authors:** Patrick L Jacobs

**Affiliations:** 1 Superior Performance Research, Miami, Florida, USA

## Background

CrossFit is a form of structured circuit exercise with tens of thousands of participants worldwide. However, little is known regarding nutritional strategies that may enhance CrossFit performances and/or recovery. Few, if any, pre-workout products have been properly examined in regards to CrossFit using double blinded, placebo-controlled research designs. The primary purpose of this study was to determine the acute effects of wodFuel® (wodFuel, Inc; Coral Springs, FL) on performance of the CrossFit exercise series, the Cindy. A secondary purpose of the study was to examine the acute effects of wodFuel® on resting hemodynamics.

## Methods

Nineteen recreationally active men (n=10) and women (n=9) with at least six months experience with CrossFit training voluntarily participated in this study. All study participants completed two test sessions of the Cindy exercise routine with one week between sessions. Each test session was performed with one of two supplement conditions applied in randomized order in a double blind fashion. One supplement condition was wodFuel®, a preworkout product specifically designed for the CrossFit setting, with ingredients including beta alanine, grape seed extract, brown rice extract, natural caffeine, minerals and vitamins. The second study condition was a look-alike, taste-alike placebo condition consisting of cellulose. Study participants were directed to report to the training facility in the morning after a 12 hour fast. Following a 30 minute rest period, baseline values of heart rate, blood pressures and psychological mood were assessed using an automated system (Dynamap 1846SX; Critikon Company LLC, Tampa, Fla). Study participants were then provided the respective supplement in powdered form mixed in eight ounces of water and directed to sit quietly in the active training environment. The hemodynamic measures (HR, BPs) were repeated twenty minutes following ingestion of the supplement. Participants then completed the Cindy exercise routine which involves the performance of as many exercise rounds of 5 pullups, 10 pushups and 15 bodyweight squats, as possible. The study participants completed as many rounds of Cindy as possible in a 20 minute period. The number of total repetitions completed in each study condition served as the primary study outcome. One way ANOVA for repeated measures was used to compare the total repetitions completed between testing conditions. Two-way ANOVAs for repeated measures was used to examine the pre- and post-supplementation values of HR and BPs. The accepted level of statistical significance was set at p < 0.05for all analyses. Consent to publish the results was obtained from all participants.

## Results

Analysis indicated that the wodFuel® test condition produced a statistically greater number of repetitions of Cindy (503.5±83.9 repetitions) compared with PL (477.5±75.6 repetitions) p < 0.05. There were no significant differences detected between pre- and post-supplementation values of HR or BPs for either wodFuel® or placebo, nor between study conditions at any time point.

## Conclusions

These findings indicate performance of the common CrossFit exercise routine is significantly enhanced with the commercial pre-workout product, wodFuel®. These results also suggest that wodFuel® may allow superior training and competitive performance without additional hemodynamic stresses.

